# Identifying potential biomarkers for early evaluating mechanical compression injuries to skeletal muscle through proteomic analysis: A rat model

**DOI:** 10.1371/journal.pone.0324706

**Published:** 2025-05-27

**Authors:** Huiyang Jia, Heng Zhang, Yan Liu, Jialiang Guo, Wei Chen, Yingze Zhang, Marius M. Scarlat, Lin Liu, Zhiyong Hou

**Affiliations:** 1 Department of Orthopaedic Surgery, Hebei Medical University Third Hospital, Shijiazhuang, China; 2 Key Laboratory of Biomechanics of Hebei Province, Shijiazhuang, Hebei Province, China; 3 Key Laboratory of Precise Assessment, Diagnosis, and Treament of Soft Tissue Injury of Hebei Province, China; 4 Department of Endocrinology, Hebei Medical University Third Hospital, Shijiazhuang, Hebei Province, China; 5 Clinique Chirurgicale St Michel, Toulon, France; University of California, Davis, UNITED STATES OF AMERICA

## Abstract

The skeletal muscle is highly susceptible to injury in daily life. Severe skeletal muscle injuries often result in incomplete regeneration, leading to functional impairment. In clinical practice, understanding the extent of skeletal muscle injury in limb trauma patients is crucial for selecting treatment modalities and assessing prognosis. Currently, there is a lack of specific indicators for evaluating the severity of mechanical skeletal muscle injury. Therefore, the aim of this study is to develop biomarkers for the early evaluation of different degrees of skeletal muscle injury. A rat model of skeletal muscle mechanical compression injury was established with varying degrees of injury severity, one control group, and two compression groups (Mild Injury and Severe Injury Group). LC-MS/MS-4D-DIA quantitative proteomics technology was used to detect the plasma proteome profile of rats in different injury groups at 3 hours post-injury, followed by bioinformatics analysis for data decoding. Rats in the mild and severe injury groups exhibited completely different degrees of injury and prognosis. The proteomic results of the plasma revealed that the relative quantification of 37 proteins increased along with the increase in injury, while 2 proteins decreased. These differentially expressed proteins (DEPs) included not only muscle-specific structural proteins but also metabolic-related proteins that might play crucial roles in tissue injury control, repair, and regeneration. Overall, the study has identified several potential protein biomarkers that can distinguish different degrees of skeletal muscle injury at an early stage. These protein biomarkers may be further developed to help clinicians identify patients with varying degrees of skeletal muscle injury, paving the way for personalized treatments.

## Introduction

Skeletal muscle is the most abundant tissue in the body, and it accounts for 40%-50% of total body mass in healthy individuals, playing a crucial role in maintaining quality of life [1]. However, it is also susceptible to damages in daily production and life, and its severity of injuries varies [2]. Skeletal muscles have a certain degree of self-healing ability after injury. However, when the damage exceeds their regenerative capacity, complete recovery cannot be achieved. Severe skeletal muscle injuries often result in incomplete regeneration, leading to functional impairments that significantly impact patients’ daily work and life abilities. In clinical practice, understanding the degree of skeletal muscle injury in patients with limb trauma is crucial for selecting treatment modalities and assessing prognosis. Currently, there is still a lack of systematic evaluation methods and indicators for assessing the degree of skeletal muscle injury. Therefore, understanding the molecular characteristics of skeletal muscle injury at different degrees in peripheral blood can provide strong support for the development of early diagnostic biomarkers for assessment on skeletal muscle injury, and for the further development of targeted clinical therapies for skeletal muscle repair.

Before establishing systematic screening procedures for the genome, transcriptome, proteome, and metabolome, targeted biomedical investigations are a common approach to identify disease biomarkers [3]. The existing classical serum protein markers related to muscle tissue breakdown and fiber injury include creatine kinase, lactate dehydrogenase, myoglobin, aldolase, and aspartate aminotransferase, among which creatine kinase is the most commonly used serum marker for muscle injury [4]. However, these established enzymes lack high diagnostic reliability, as creatine kinase can still increase after regular exercise [5]. They also cannot effectively differentiate the severity of the injury. With the rapid development of life science technologies, especially high-throughput techniques, the detection methods for biomarkers after injury have been greatly improved [6–9]. Liu et al. [7] investigated the changes in the rat muscle proteome during the phases of damage, repair, and early remodeling following impact injury. Li et al. [9] applied gene chips and bioinformatics analysis to describe the molecular spectrum changes after skeletal muscle contusion. However, there have been no reports on the characteristics of early plasma proteomics after varying degrees of mechanical compression injury in rat skeletal muscle.

Therefore, this study aims to establish a rat model of mechanical compression injury in skeletal muscle. Plasma protein expression profiles of different injury groups were detected using LC-MS/MS-4D-DIA quantitative proteomics technique. Bioinformatic analysis was performed to decode the data, aiming to explore specific biomarkers suitable for early diagnosis of skeletal muscle injuries in varying degrees. These biomarkers could assist clinicians in evaluating the levels of skeletal muscle injury for more personalized treatment therapies, thereby improving patient outcomes and quality of life. This study could also provide solid scientific foundation for further exploration of the underlying mechanisms of the injury, repair and regeneration of skeletal muscle.

## Materials and methods

### Animals and injury model

All experiments received approval from the Animal Care and Use Committee of the Third Hospital of Hebei Medical University (Z2022-009-1) and were conducted following the National Institutes of Health Guidelines for the Care and Use of Laboratory Animals. Fifty-five Sprague-Dawley (SD) rats (male, 10–11 weeks old, weighing between 350-360g) were procured from Beijing Huafukang Biotechnology Co., Ltd. The rats were housed under controlled conditions, including standard chow, 12-hour light/12-hour dark cycles, and maintained at a constant temperature (22–24 °C) and humidity (50–65%). Experiments were consistently conducted at similar times to mitigate any potential circadian rhythm effects on the rats.

**Fig 1:**
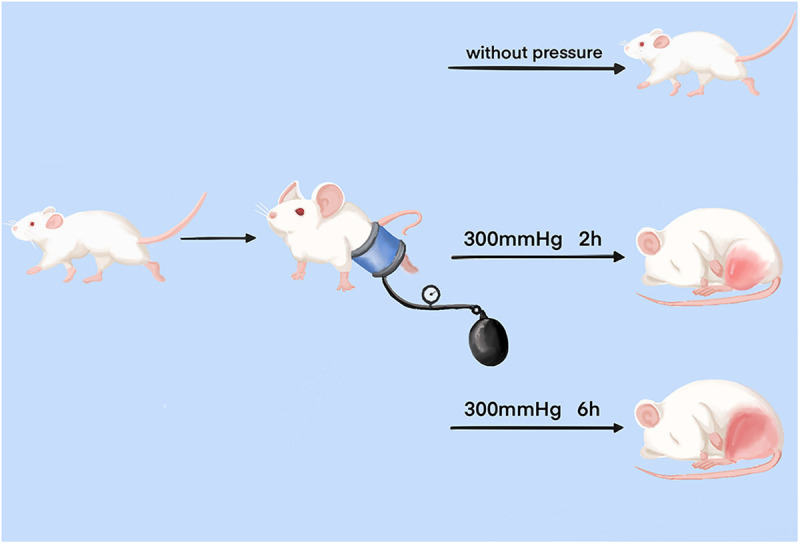
Schematic diagram of the process for establishing skeletal muscle mechanical compression injury models with varying degrees of severity.

A novel compression device, consisting of a pressure gauge cuff embedded in a rigid plastic tube as described earlier [10], was used to induce varying degrees of mechanical compression injury in the lower leg skeletal muscle of the rat. This simulated crush injuries of varying degrees in the limbs of clinical patients. This device cuff could fully envelop the entire lower leg of the rat. After a 7-day acclimatization period, fifty-five rats were randomly divided into three groups, namely the control group (C), the mild injury group (M), and the severe injury group (S). Control group consisted of 5 rats that were only anesthetized and fixed in the compression device without pressure. The Mild injury group included 25 rats subjected to a pressure of 300 mmHg for 2 hours. Similarly, the Severe injury group comprised 25 rats exposed to a pressure of 300 mmHg for 6 hours (Fig 1). In the injury group, 25 rats were euthanized at five time points after injury: 3 hours, 3 days, 7 days, 14 days, and 28 days, with 5 rats at each time point. All tested samples were collected accordingly.

SD rats were anesthetized using 3% isoflurane for induction, followed by maintenance with 1.5% isoflurane. Once fully anesthetized, the right lower limb was shaved for modeling purposes. Postoperative analgesia was administered with 1% lidocaine infiltration and continued until sampling time. After the injury, the rats were transferred to clean cages with unrestricted access to food and water. At the conclusion of the experiment, all animals were euthanized by intraperitoneal injection of 200 mg/kg sodium pentobarbital and 10mg/mL lidocaine. Subsequently, all animal carcasses were uniformly incinerated.

### Sample collection and histological examination

**Fig 2:**
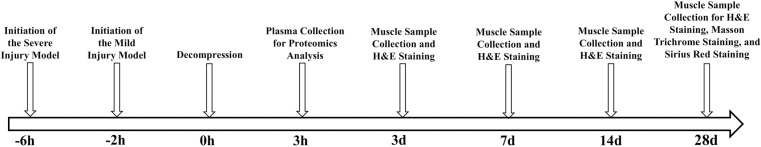
Experimental design.

The experimental procedure was shown in Fig 2. In order to obtain blood biomarkers that could be used to assess varying degrees of muscle injury in the early stages of injury, blood samples were collected by cardiac puncture from three groups of rats (n = 5) 3 hours post-injury. After centrifugation (10 minutes, 3000rpm), the plasma samples were rapidly frozen in liquid nitrogen and stored in a -80°C freezer for subsequent proteomic analysis. At this point, tibialis anterior (TA) muscles from the control group rats were collected as a normal control group.

At 3d, 7d, 14d, and 28d, muscle samples of the injured side tibialis anterior (TA) from the injury group were collected for histological examination. The muscles were fixed in 10% neutral buffered formalin (P110, Solarbio, China) for 48 hours and then transferred to 70% ethanol (Yongda Chemical Reagent Company, China). The TA muscles were embedded in paraffin and sectioned into 4 μm thick slices. Muscle sections obtained at 3 days, 7 days, 14 days, and 28 days post-injury were stained with Hematoxylin and Eosin (H&E) to assess the extent of muscle injury and regeneration. Muscle sections obtained at 28 days post-injury were stained with Masson trichrome and Sirius Red (Servicebio, Wuhan, China) to assess the degree of fibrosis.

Stained sections were analyzed at 200x magnification using a Nikon Eclipse Ci-L microscope (Japan). The determination of the proportion of injured muscle fibers was conducted according to the scheme outlined by McCormack et al [11]. Injured myofibers are defined as ragged cellular edges, vacuolation, Inflammatory cell infiltration, or rhabdomyolysis [12]. Regenerative myofibers were identified as those containing central nuclei [13]. At 7 days and 14 days post-injury, five random fields were selected from each sample at 20x objective lens magnification to quantify the total number of regenerating myofibers, and the short-axis diameter of each regenerating myofiber was measured, as described previously [14]. At 28 days post-injury, five random fields were selected from each sample at 20x objective lens magnification, and the average cross-sectional area of myofibers in each group was quantified using Image-Pro Plus 6.0 software [15]. At 28 days post-injury, five randomly selected regions from each sample stained with Masson trichrome and Sirius Red at 20x objective lens magnification were quantitatively measured for fibrosis using Image-Pro Plus 6.0 software.

### Protein extraction

Initially, cellular debris was eliminated from the plasma sample by centrifugation at 12,000 g at 4 °C for 10 minutes. Subsequently, the supernatant was transferred to a new centrifuge tube. Finally, the protein concentration was assessed using a BCA kit (Thermo Fisher Scientific, USA) following the manufacturer’s guidelines.

### Trypsin digestion

50 µL of centrifuged high-speed blood sample were transferred to pre-washed magnetic nanoparticles (PTM-00F13303, sourced from PTM Bio, Hangzhou Jingjie Biotechnology Co., Ltd.), and incubated at 37°C for 1 hour on a constant temperature mixer at 1200 rpm. After the incubation, the magnetic beads were washed three times with washing buffer. Subsequently, 70 μL of enzymatic digestion buffer was added to the beads. After mixing, they were heated at 95°C for 10 minutes. Following the heating process, the samples were allowed to return to room temperature. Then, trypsin was added to a final concentration of 20 ng/μL, and the mixture was incubated at 37°C overnight for enzymatic digestion. Dithiothreitol (DTT) was added to achieve a final concentration of 5 mM, and the mixture was reduced at 56°C for 30 minutes. Subsequently, iodoacetamide (IAM) was added to achieve a final concentration of 11 mM, and the mixture was incubated at room temperature in the dark for 15 minutes. Finally, desalination was performed according to the C18 ZipTips (MilliporeSigma, Germany) manual, followed by vacuum freeze-drying for subsequent liquid chromatography tandem-mass spectrometry (LC-MS/ MS) analysis.

### LC-MS/MS analysis

The mobile phase consisted of solvent A (0.1% formic acid, 2% acetonitrile in water) and solvent B (0.1% formic acid, 90% acetonitrile in water). The tryptic peptides were dissolved in solvent A and directly injected onto a homemade reversed-phase analytical column (25 cm length, 100 μm i.d.). Peptides were eluted with the following gradient: 0–16 min, 6%-20% B; 16–24 min, 20%-32% B; 24–27 min, 32%-80% B; 27–30 min, 80% B, all at a constant flow rate of 500 nl/min using an EASY-nLC 1200 UPLC system (Thermo Fisher Scientific, USA). The separated peptides were analyzed in an Orbitrap Exploris 480 mass spectrometer equipped with a nano-electrospray ion source (Thermo Fisher Scientific, USA). The electrospray voltage applied was 2,100 V. Precursor ions and fragments were detected in the Orbitrap detector. The full MS scan resolution was set to 30,000 over a scan range of 350–1,050 m/z. The MS/MS scan was triggered by precursor ions with a minimum m/z of 200.0 and a resolution of 450,000. HCD fragmentation was carried out at normalized collision energies (NCE) of 25%, 30%, and 35%. The automatic gain control (AGC) target was set to 3E6, with a maximum injection time set to Auto.

### Database search

The DIA data were analyzed using the DIA-NN search engine (v.1.8). Tandem mass spectra were searched against Rattus_norvegicus_10116_PR_20231121.fasta (47943 entries) concatenated with reverse decoy database. Trypsin/P was designated as the cleavage enzyme with allowance for up to 1 missing cleavage. Fixed modifications included excision on N-terminal Met and carbamidomethyl on Cys. False Discovery Rate (FDR) was adjusted to < 1%.

### Bioinformatics methods

Gene ontology (GO) and Kyoto Encyclopedia of Genes and Genomes (KEGG) pathway analyses were performed on differentially expressed proteins.

Gene Ontology analysis is a bioinformatic analysis method that organically links various information about genes and gene products (such as proteins) to provide statistical information [16]. The GO annotation process entailed extracting GO IDs from identified proteins using eggnog-mapper software based on the EggNOG database. Subsequently, functional classification annotation analysis was conducted on the proteins based on cellular components, molecular functions, and biological processes. For each category, a two-tailed Fisher’s exact test was used to test the enrichment of differentially expressed proteins relative to all identified proteins. A corrected p-value <0.05 was considered significant.

The Kyoto Encyclopedia of Genes and Genomes (KEGG) integrates the currently known protein-protein interaction network information. KEGG pathways mainly include metabolism, genetic information processing, environmental information processing, cellular processes, human diseases, and drug development. We annotated protein pathways based on the KEGG pathway database, and performed BLAST comparisons (blastp, evalue ≤ 1e-4) for the identified proteins. For each sequence, the annotation was based on the top-scoring comparison result. We conducted pathway enrichment analysis for differentially expressed proteins using Fisher’s exact test (Fisher’s exact test), with the identified proteins as the background. A p-value < 0.05 was considered significant.

After aligning differentially expressed proteins with the STRING database, protein-protein interaction relationships were obtained (confidence score > 0.7). A visual network was constructed using Cytoscape 3.10.0, and the top ten hub genes were identified using the cytoHubba plugin (MCC algorithm).

### Statistical analysis

Python (version 3.7.6) was used to perform the statistical analyses. A one-way ANOVA was performed to check for significant differences between the groups. After finding significance, Tukey’s HSD test was used for post-hoc analysis, with p < 0.05 indicating statistical significance.

## Results

### Histopathological staining was used as the gold standard to assess varying degrees of injury, regeneration and fibrosis among the animal models in each group

**Fig 3:**
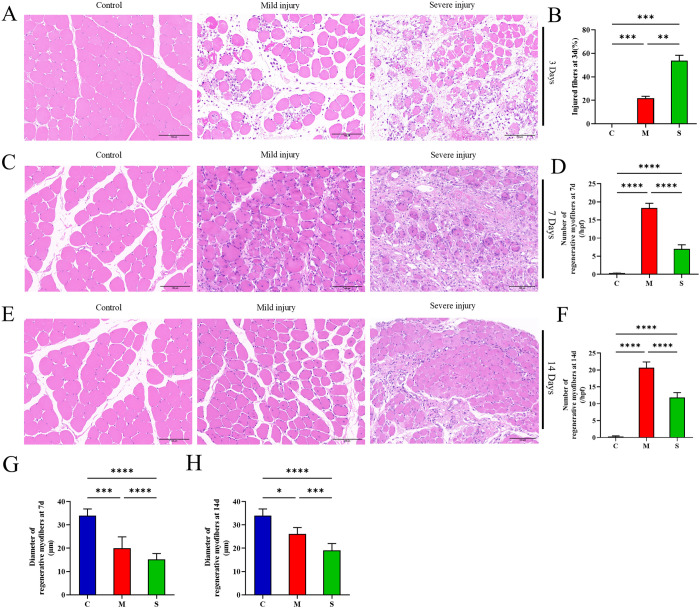
H&E staining of the tibialis anterior muscle in rats at 3, 7, and 14 days after mechanical compression injury of varying degrees. (A) Typical H&E staining images of tibialis anterior muscle 3 days post-injury. (B) Percentage of injured myofibers at 3 days post-injury. (C) Representative images of muscle regeneration at 7 days post-injury. (D) Quantitative analysis of the number of regenerated muscle fibers in each group at 7 days post-injury. (E) Representative images of muscle regeneration at 14 days post-injury. (F) Quantitative analysis of the number of regenerated muscle fibers in each group at 14 days post-injury. (G) Quantitative analysis of the diameter of regenerated muscle fibers in each group at 7 days post-injury. (H) Quantitative analysis of the diameter of regenerated muscle fibers in each group at 14 days post-injury. All data are presented as mean ± standard deviation. N = 5.

**Fig 4:**
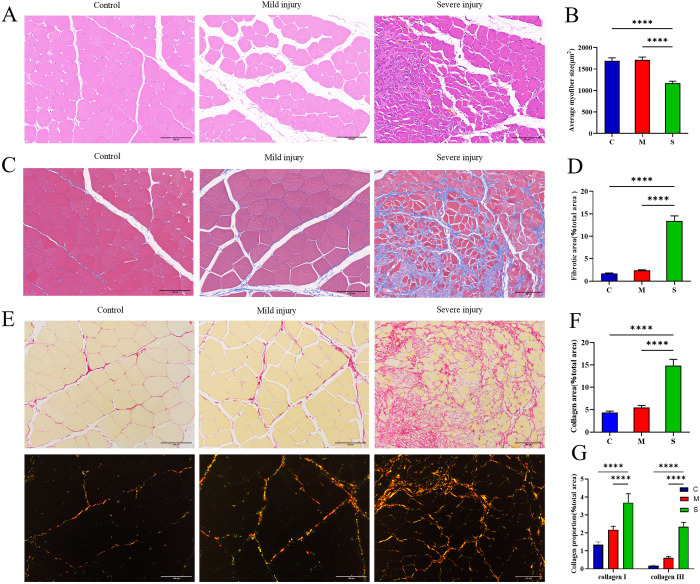
Histopathological staining of the tibialis anterior muscle in rats at 28 days after mechanical compression injury of varying degrees. (A) Representative images of muscle regeneration, as shown by H&E staining. (B) Quantitative analysis of the average myofiber size in each group. (C) Representative image of fibrosis in each group, as shown by Masson trichrome staining. (D) Quantitative analysis of the fibrotic area in each group. (E) Representative image of Sirius Red staining at 28 days post-injury under bright field and polarized light microscopy. (F) Quantitative analysis of collagen fiber area under bright field microscopy. (G) Quantitative analysis of type I and type III collagen fiber area under polarized light. All data are presented as mean ± standard deviation. N = 5.

The morphological characteristics of skeletal muscle injury in rats from each group were observed 3 days post-injury using H&E staining. As shown in Fig 3A, H&E staining revealed mild disruption of muscle fibers and moderate interstitial edema accompanied by inflammatory cell infiltration in the mild injury group. In the severely injured group, muscle fibers exhibited severe disruption accompanied by a significant infiltration of inflammatory cells. The proportion of injured fibers in the severe injury group was significantly higher than that in the mild injury group and the control group (Fig 3B). At 7 and 14 days post-injury, regenerated muscle fibers with central nuclei were observed in all injury groups. However, the number and diameter of regenerated muscle fibers in the mild injury group were both greater than those in the severe injury group (Fig 3C–3H). At 28 days post-injury, although some muscle fibers with central nuclei could still be observed in the mild injury group, the average muscle fiber area and histopathological characteristics showed no difference compared to the control group. In contrast, the average muscle fiber area in the severe injury group was significantly smaller than that of the control group, with extensive fibrosis observed. (Fig 4).

### The plasma proteomic profiles of three groups of animals exhibited significant differences

**Fig 5:**
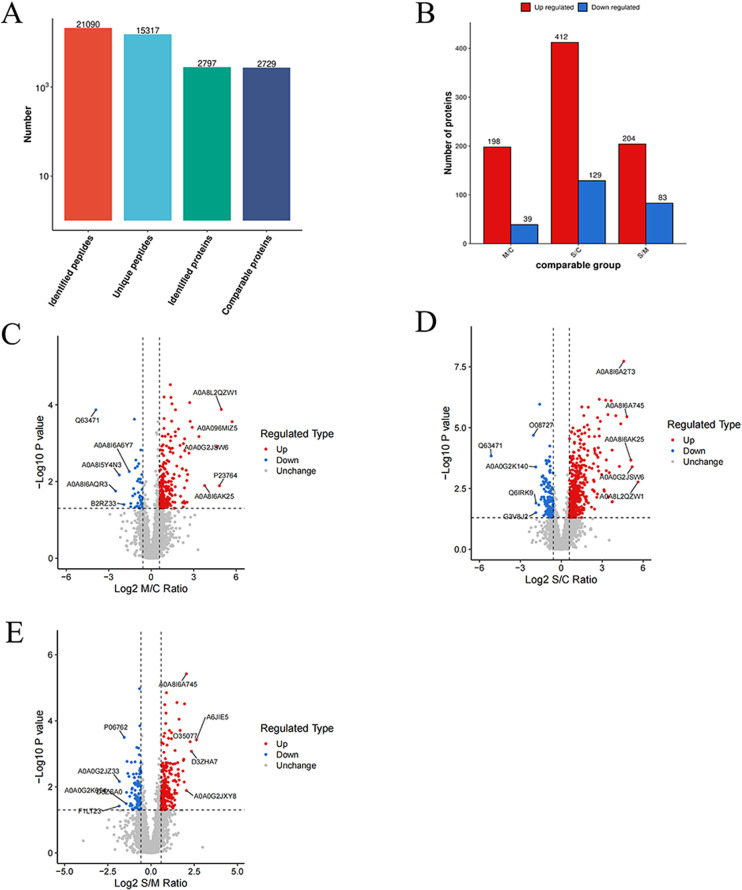
Protein identification results. (A) The total number of peptides and proteins identified. (B) Bar graph showed the number of differentially expressed proteins in pairwise comparisons among the three groups. (C-E) Volcano plots of pairwise comparisons among the three groups. (C) Comparison between the mild injury group and the control group. (D) Comparison between the severe injury group and the control group. (E) Comparison between the severe injury group and the mild injury group. Red dots represented significantly upregulated proteins, blue dots represented significantly downregulated proteins, and gray dots represented proteins with no significant difference. The names of the top 5 upregulated and downregulated proteins (ranked by absolute Log2 Ratio) were labeled in the plot.

A total of 2,797 proteins with at least one unique peptide were identified with an FDR ≤ 1% (Fig 5A). Through differential analysis, when P value was less than 0.05, fold change exceeding 1.5 was considered as significant upregulation threshold, and being less than 1/1.5 was considered as significant downregulation threshold. Compared to the C group, the M group exhibited significant differences in 237 proteins (198 upregulated, 39 downregulated), while the S group showed significant differences in 541 proteins (412 upregulated, 129 downregulated). Relative to the M group, the S group displayed significant differences in 287 proteins (204 upregulated, 83 downregulated) (Fig 5B). The volcano plot illustrated significant changes in protein expression levels among pairwise comparisons of the three groups. Red dots represented significant upregulation, blue dots represented significant downregulation, and gray dots indicated no significant difference. The top 5 upregulated and downregulated proteins (sorted by absolute Log2 Ratio values) were concurrently labeled in the plot (Fig 5C–5E).

### Thirty-nine proteins could distinguish different degrees of muscle damages

**Fig 6:**
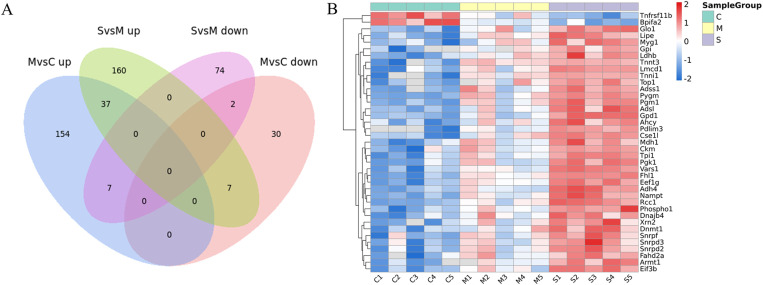
Thirty-nine proteins could distinguish different degrees of muscle damage. (A) The union of the intersection between the differentially upregulated proteins in pairwise comparisons MvsC and SvsM, and the intersection between the differentially downregulated proteins in pairwise comparisons MvsC and SvsM, yielded a total of 39 proteins. (B) a quantitative heatmap of the intersecting proteins.

Intersection protein analysis was performed to identify proteins with significant differential expression capable of distinguishing various degrees of muscle damage. The relative quantification values of 37 proteins increased with the severity of muscle damage, while those of 2 proteins decreased as the degree of muscle damage escalated (Fig 6A). The heatmap depicted the relative expression levels of 39 proteins across the three groups and illustrated their clustering relationships. Each row represented a differentially expressed protein, while each column represented a sample. Red indicated high expression, blue indicated low expression, and gray indicated non-quantifiable levels in the corresponding sample (Fig 6B). The detailed information on differentially expressed proteins (DEPs) along with fold changes is listed in Table 1.

**Table 1:** The 39 differentially expressed proteins (DEPs) were capable of distinguishing different degrees of muscle injury and their fold changes.

Accession Number	Gene	Protein Description	MvsC FC	SvsM FC	SvsC FC
A0A0H2UHY9	Tnnt3	Troponin T3, fast skeletal type	117.19	2.07	242.65
A0A096MIZ5	Tnni1	Troponin I1, slow skeletal type	52.62	2.99	157.26
A0A8I6A2T3	Pygm	Alpha-1,4 glucan phosphorylase	10.36	2.30	23.83
A0A8I6A745	Fhl1	Four and a half LIM domains 1	6.73	4.15	27.93
A0A0G2JSM3	Pdlim3	PDZ and LIM domain 3	6.35	3.22	20.44
A6JRM5	Pgm1	Phosphoglucomutase 1	5.16	3.09	15.96
M0R629	Adss1	Adenylosuccinate synthetase isozyme 1	5.09	2.10	10.69
G3V8R5	Lipe	Hormone-sensitive lipase	4.84	2.73	13.22
A0A0G2JSP8	Ckm	creatine kinase	4.79	2.79	13.39
A0A0G2K3V7	Top1	DNA topoisomerase I	3.95	1.74	6.87
Q6AYF2	Lmcd1	LIM and cysteine-rich domains 1	3.23	2.69	8.71
P42123	Ldhb	L-lactate dehydrogenase B chain	2.90	2.08	6.03
A0A8I6A243	Gpi	Glucose-6-phosphate isomerase	2.87	1.83	5.24
A0A8L2QAQ2	Tpi1	Triosephosphate isomerase	2.66	2.41	6.42
A0A0G2K7T1	Rcc1	Regulator of chromosome condensation 1	2.63	2.62	6.88
O35077	Gpd1	Glycerol-3-phosphate dehydrogenase [NAD (+)], cytoplasmic	2.62	4.80	12.58
A0A0G2K0I3	Nampt	Nicotinamide phosphoribosyltransferase	2.56	2.62	6.73
A0A8I6A721	Mdh1	Malate dehydrogenase	2.44	1.94	4.73
P16617	Pgk1	Phosphoglycerate kinase 1	2.26	2.44	5.52
A0A8I6A1F9	Armt1	Sugar phosphate phosphatase	2.25	2.27	5.11
A0A8I5ZMZ1	Fahd2a	Fumarylacetoacetate hydrolase domain containing 2A	2.09	1.53	3.19
D3ZPR0	Cse1l	Exportin-2	2.08	1.85	3.86
Q5XIP0	Dnajb4	DnaJ heat shock protein family (Hsp40) member B4	2.07	1.68	3.47
Q68FR6	Eef1g	Elongation factor 1-gamma	1.98	1.67	3.30
A0A8I6A6B0	Phospho1	Phosphoethanolamine/phosphocholine phosphatase 1	1.93	1.95	3.77
A0A8I5ZTN5	Adsl	Adenylosuccinate lyase	1.83	2.23	4.09
A0A8I5ZUU8	Xrn2	5’-3’ exoribonuclease	1.74	1.60	2.79
F7FB70	Snrpd2	Small nuclear ribonucleoprotein Sm D2	1.72	1.56	2.69
D4AAT4	Snrpf	Sm protein F	1.70	1.62	2.75
Q6P7Q4	Glo1	Lactoylglutathione lyase	1.67	1.53	2.56
P10760	Ahcy	Adenosylhomocysteinase	1.66	1.74	2.89
F1LQT9	Dnmt1	DNA (cytosine-5)-methyltransferase	1.64	1.66	2.72
A0A8I6B5U9	Eif3b	Eukaryotic translation initiation factor 3 subunit B	1.61	1.62	2.61
Q641W2	Myg1	MYG1 exonuclease	1.61	1.59	2.56
A0A8I6AI37	Snrpd3	Small nuclear ribonucleoprotein Sm D3	1.56	1.67	2.61
Q04462	Vars1	Valine--tRNA ligase	1.54	1.74	2.68
Q7TQ90	Adh4	Alcohol dehydrogenase class-3	1.52	2.10	3.18
O08727	Tnfrsf11b	Tumor necrosis factor receptor superfamily member 11B	0.47	0.51	0.24
Q63471	Bpifa2	BPI fold-containing family A member 2	0.07	0.43	0.03

### GO and KEGG analysis of the differential expression of proteins

**Fig 7:**
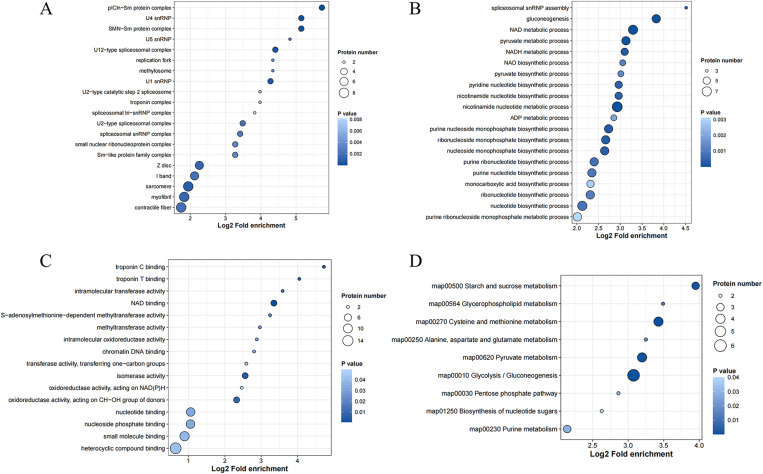
GO and KEGG analysis of DEPs. (A) Cellular component of DEPs. (B) Biological processes of DEPs. (C) Molecular function of DEPs. (D) KEGG pathways. The x-axis represented the fold enrichment of functional enrichment after Log2 transformation, with larger values indicating higher enrichment levels. The color of the dots indicated the significance of enrichment P value, with darker blue indicating stronger enrichment significance. The size of the dots represented the number of differential proteins, with larger dots indicating a greater number of differential proteins.

GO and KEGG enrichment analysis was performed on the 39 proteins that could be used to distinguish different degrees of muscle injury. Gene Ontology (GO) enrichment analysis outlined the functions of the aforementioned DEPs. In terms of cellular components, DEPs primarily involved contractile fibers, myofibrils, sarcomeres, I-bands, and Z-discs (Fig 7A). DEPs mainly pertained to gluconeogenesis, NAD metabolism, pyruvate metabolism, nicotinamide nucleotide metabolic process and nucleotide biosynthesis biological processes (Fig 7B). Molecularly, DEPs were primarily associated with binding to heterocyclic compounds, small molecules, nucleotides, and NAD (Fig 7C).

Based on KEGG-based functional enrichment analysis, DEPs were significantly enriched in multiple processes (Fig 7D), including glycolysis/gluconeogenesis, pyruvate metabolism, cysteine and methionine metabolism, as well as starch and sucrose metabolism.

The GO and KEGG analysis of DEPs indicated that some skeletal muscle-specific structural proteins and metabolism-related proteins were significantly upregulated. These proteins might leak into the blood as a result of skeletal muscle rupture, including Fast skeletal muscle Troponin T, slow skeletal type Troponin I1, Four and a half LIM domains 1, PDZ and LIM domain 3, Hormone-sensitive lipase, Alpha-1,4 glucan phosphorylase, cytoplasmic glycerol-3-phosphate dehydrogenase, Phosphoglucomutase 1, Nicotinamide phosphoribosyltransferase, and Glucose-6-phosphate isomerase.

### Protein–protein interaction (PPI) network of the DEPs

**Fig 8:**
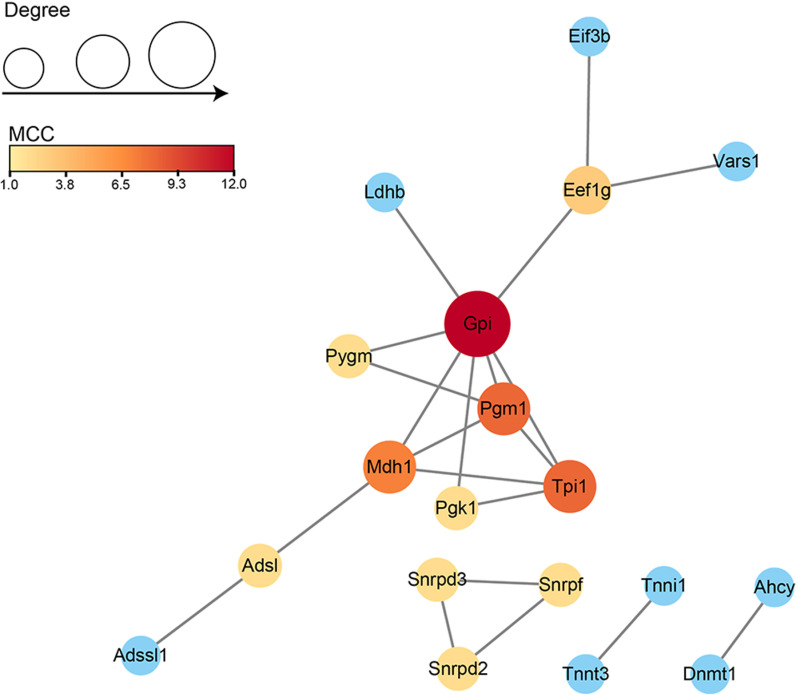
Protein-protein interaction network construction. The color represented the MCC value of DEPs, with darker colors indicating higher MCC values. The size of the node represented the degree, where larger nodes indicated higher degrees. Generally, proteins with higher degrees or MCC values were more likely to be key proteins.

In general, proteins with high degree or MCC are more likely to be key proteins. Glucose-6-phosphate isomerase (Gpi) exhibited the highest degree and MCC value. The functions of other proteins in the PPI network were primarily involved in carbohydrate metabolism (Fig 8). These data also indicated dramatic changes in metabolic-related processes during muscle injury. The significantly altered proteins may serve as biomarkers for assessing muscle injury.

## Discussion

This study investigated the expression of plasma proteins in rat models of skeletal muscle mechanical compression injuries of varying degrees of severity. We identified a total of 2,797 proteins with at least one unique peptide. Among these, the relative quantitation of 37 proteins increased with the severity of muscle injury, while the relative quantitation of 2 proteins decreased with the severity of muscle injury. These differentially expressed proteins (DEPs) included not only muscle-specific structural proteins but also metabolic-related proteins that might play crucial roles in tissue injury control, repair, and regeneration. These differentially expressed proteins may serve as specific biomarkers for the early diagnosis of skeletal muscle injuries of varying degrees in the future.

We established a rat model of skeletal muscle mechanical compression injury with varying degrees of severity. The histopathological results showed that the proportion of injured fibers in the mild injury group was significantly lower than that in the severe injury group. The mild injury group exhibited more regenerated fibers with larger fiber diameters, and by day 28, the fiber size had returned to normal, indicating stronger regenerative capacity in the mild injury group. In contrast, the severe injury group demonstrated slow regeneration, poor quality of regenerated fibers, and extensive fibrosis characterized by the predominance of mature type I collagen fibers [17]. At 28 days post-injury, the gait of rats in the mild injury group was nearly normal, while rats in the severe injury group exhibited muscle contractures and limping. The results laid a solid foundation for the further exploration of underlying mechanism of musculoskeletal muscle injury with candidate biomarkers.

In our proteomic results, the most significant DEPs were fast skeletal muscle Troponin T and slow skeletal type Troponin I1. Skeletal muscle troponin is a protein complex located within muscle fibers, playing a significant role in regulating the process of muscle contraction. The troponin complex consists of three subunits: troponin C (TnC), which binds calcium ions (Ca2+); troponin I (TnI), the inhibitory subunit; and troponin T (TnT), which binds to tropomyosin (Tm). TnC has two isoforms, one found in fast skeletal muscle and the other in cardiac and slow skeletal muscles. In contrast, TnI and TnT, which evolved from a TnI-like ancestor, each have three isoforms specific to cardiac, slow skeletal, and fast skeletal muscles [18,19]. Fast skeletal muscle TnT (fsTnT) is found exclusively in fast-twitch fibers and undergoes complex N-terminal alternative splicing, leading to a decrease in molecular weight during postnatal development [19,20]. Borkowski et al. [21] reported a significant increase in serum concentrations of skeletal muscle TnT among marathon participants and participants in a 100-kilometer adventure race, with the marathon group showing a 127% increase and the adventure race group showing a 113% increase. Further research is needed to evaluate the diagnostic value of fast skeletal muscle TnT for mechanical skeletal muscle compression injuries. The cardiac isoform of troponin I (cTnI) is exclusively expressed in cardiac muscle and is currently regarded as the gold standard for detecting myocardial injury [22]. The fast skeletal troponin I isoform (fsTnI) and the slow skeletal troponin I isoform (ssTnI) are exclusively present in adult fast-twitch and slow-twitch skeletal muscle fibers, respectively [23]. The majority of human limb skeletal muscles contained a combination of both slow (type I) and fast (type II) muscle fibers. Chapman et al. measured the changes in serum concentrations of fast and slow skeletal muscle troponin I (fsTnI and ssTnI, respectively) following maximal eccentric contractions. They observed a significant increase in serum fsTnI levels after exercise, while ssTnI remained unchanged [24]. Westfall et al. [25] found that slow skeletal muscle TnI enhances the sensitivity of contraction activation to Ca2+ in adult rat cardiomyocytes under physiological and acidic pH conditions. This indicates that slow skeletal muscle TnI plays a key role in mediating skeletal muscle resistance to acidosis[19]. Therefore, the slow skeletal troponin I isoforms (ssTnI) may serve as specific biomarkers for skeletal muscle mechanical compression injuries.

Four and a half LIM domains 1 (Fhl1) was first identified in skeletal muscle by Morgan et al. [26], initially termed Skeletal Muscle LIM protein (SLIM1). Fhl1 is most abundant in skeletal muscle, and abnormalities in the Fhl1 gene have been identified as pathogenic factors in various muscle disorders, such as X-linked myopathy [27], myofibrillar myopathy, muscular dystrophy [28], inflammatory myopathy [29] and reducing body myopathy [30]. Over the past decade, Fhl1 has been found to play a dual role in cancer progression [31]. However, to our knowledge, there are no reports regarding the diagnostic value of Fhl1 for skeletal muscle injury. In our study, the relative expression level of Fhl1 in plasma increased significantly with the severity of muscle injury, suggesting its potential as a biomarker for distinguishing different degrees of muscle injury in the future. PDZ and LIM domain 3 (Pdlim3) is highly expressed in skeletal muscle and is thought to play a role in organizing actin filament arrays in muscle cells [32,33]. It was previously reported that Pdlim3 binds the spectrin-like repeats of α-actinin-2, co-localizing with α-actinin-2 at the Z lines of skeletal muscle [34]. Gan et al. [35] found that Pdlim3 serves as a specific biomarker for endometriosis. Lak et al. [36] identified Pdlim3 as one of the biomarkers for rhabdomyosarcoma and found its correlation with clinical outcomes. Wang et al. [37] discovered that genetic polymorphisms in the Pdlim3 gene increased the risk of idiopathic dilated cardiomyopathy. Other studies have also indicated associations of Pdlim3 with atrial fibrillation [38], myofibrillar myopathy [39], early regional metastasis of tongue cancer [40], and invasive bladder urothelial carcinoma involving the muscularis propria [41]. This is the first time that Pdlim3 has been linked to skeletal muscle mechanical crush injuries. As a result, it could play a significant role in diagnosing mechanical muscle injuries.

Significant enrichment of DEPs in metabolic pathways was observed in the study. Hormone-sensitive lipase (HSL) is an intracellular enzyme that catalyzes the hydrolysis of a range of lipid substrates, such as triglycerides, diglycerides, monoglycerides, cholesterol esters, retinol esters, and other lipid compounds [42]. HSL activity is carefully controlled by neuronal and hormonal signals in response to energy needs, with regulation occurring through reversible serine phosphorylation mediated by protein kinase A and cAMP [43]. HSL is activated by cAMP-dependent PKA under adrenergic/noradrenergic stimulation [42]. Phosphorylation of HSL enhances its hydrolytic activity, facilitates its translocation from the cytoplasm to lipid droplet surfaces, and promotes the breakdown of stored triglycerides [44]. Moreover, insulin-stimulated adipocytes inhibit HSL, leading to a decrease in the release of free fatty acids and glycerol [42]. Similar to HSL, the enzyme Alpha-1,4 glucan phosphorylase (Pygm), which catalyzed glycogen breakdown, also increased gradually with the severity of mechanical compression injury. Langfort et al. [45] found that electrical stimulation-induced skeletal muscle contractions can lead to increased activity of HSL and glycogen phosphorylase. They believed that the increase in the activity of these two enzymes is regulated in parallel by contraction-induced and hormonal mechanisms, allowing for the simultaneous recruitment of all major energy stores in both muscle exterior and interior. The relative quantification of cytoplasmic glycerol-3-phosphate dehydrogenase (Gpd1) also significantly increased. It catalyzes the oxidation of glycerol-3-phosphate (G3P) to dihydroxyacetone phosphate (DHAP) for gluconeogenesis. Furthermore, Phosphoglucomutase 1 (Pgm1) also exhibited significant differences. This enzyme catalyzes the conversion of glucose-1-phosphate into glucose-6-phosphate, which is utilized in glycolytic metabolism [46]. Therefore, the elevation of these metabolic enzymes might be able to resist compression damage and promote repair by altering the energy metabolism pattern of skeletal muscle. Subsequently, these proteins leaked into the blood due to skeletal muscle rupture. Further research is needed to explore the relevant mechanisms. These proteins may serve as novel biomarkers for diagnosing mechanical compression injuries of skeletal muscle in the future.

Nicotinamide phosphoribosyltransferase (Nampt) may also be of significance in diagnosing various degrees of muscle injury. Nampt is a key rate-limiting enzyme in the NAD salvage pathway. Knockout of Nampt specifically in mouse muscle reduces the levels of NAD+ in the muscle, leading to muscle fiber degeneration and loss of physical functions resembling those of human type 2 diabetes, such as strength and endurance [47]. Research by Manickam et al. [48] also indicates that the Nampt activator P7C3 enhances the function of skeletal muscle in diabetic model mice by regulating cellular metabolism and reducing inflammatory responses. Ratnayake et al. confirmed the indispensable role of macrophage-derived Nampt in muscle regeneration [49]. Additionally, Nampt-promoted NAD+ biosynthesis can prevent muscle disuse atrophy by reversing mitochondrial dysfunction [50]. Given Nampt’s significant role in improving muscle function and promoting regeneration, its increased expression may be a specific response of muscles to injury.

We constructed a potential protein-protein interaction network using the STRING 11.5 database. In addition to the aforementioned DEPs, Glucose-6-phosphate isomerase (Gpi) exhibited the highest degree and MCC value. Glucose-6-phosphate isomerase plays a crucial role in glycolysis, catalyzing the interconversion between glucose-6-phosphate and fructose-6-phosphate. As is well-known, skeletal muscles can secrete a large number of secretory proteins, including Gpi [51]. It can play a significant role as both angiogenic and neurotrophic factors [51,52]. Gpi’s involvement in angiogenesis is linked to the hypoxia-induced upregulation of vascular endothelial growth factor (VEGF) [52]. In neural precursor cells, Gpi can promote neurodifferentiation [51]. Additionally, Gpi also has a role in reducing oxidative stress [53]. In summary, the increased expression of Gpi may play a crucial role in the control, repair, and regeneration of skeletal muscle injuries through its potential effects in reducing oxidative stress, promoting angiogenesis. Gpi may also serve as a novel specific biomarker for injury diagnosis.

It is worth noting that fibrosis was present in the skeletal muscle of the severe injury group, suggesting that the 160 proteins significantly upregulated between the severe and mild injury groups may have a potential association with fibrosis. However, as this is not the focus of the current study, further discussion is not included here. Relevant information on these proteins is presented in the S1 File and S2–S4 Figs.

This study also has some limitations. Firstly, the sample size of experimental animals is small, which may not completely eliminate individual biases. Secondly, we have not validated the candidate biomarkers, nor have we studied the diagnostic thresholds of candidate biomarkers due to sample size limitations, so the sensitivity and specificity of these biomarkers could not be determined yet. Moreover, our results have not been validated on patients. In the future, we will increase the sample size, validate the candidate biomarkers using antibodies, verify their sensitivity and specificity, explore their diagnostic thresholds, test on trauma patients and eventually apply in clinical setting. Additionally, we will combine biomarker levels with multimodal imaging and machine learning to create a comprehensive skeletal muscle injury scoring system, thereby enabling a more accurate assessment of injury severity. Finally, our study only explored protein expression in the early stages of muscle injury. However, exploring protein expression at intermediate time points (such as 7 days and 14 days) is crucial for revealing the transitional mechanisms between regeneration and fibrosis after skeletal muscle injury, and warrants further investigation.

## Conclusion

We induced varying degrees of mechanical compression injury in the skeletal muscles of the rat’s lower leg using a novel compression device. Rats in the mild and severe injury groups exhibited completely different degrees of injury and prognosis. We conducted LC-MS/MS-4D-DIA quantitative proteomics analysis for the first time on the plasma of rats in the control, mild injury, and severe injury group. The results showed significant differences in fsTnT, ssTnI, Fhl1, Pdlim3, HSL, Pygm, Gpd1, Pgm1, Nampt, and Gpi among the three groups. These proteins may serve as novel biomarkers for the early evaluation different degrees of muscle injury in the future.

## Supporting information

S1 File The 160 differentially expressed proteins significantly elevated in the severe injury group compared to the mild injury group and their folding changes. (XLSX)

S2 Fig A quantitative Heatmap of 160 Differentially Expressed Proteins. (TIF)

S3 Fig GO and KEGG Analysis of 160 Differentially Expressed Proteins. (A) Cellular component of DEPs. (B) Biological processes of DEPs. (C) Molecular function of DEPs. (D) KEGG pathways.
(TIF)

S4 Fig Protein-Protein Interaction Network Construction for 160 Differentially Expressed Proteins. (TIF)
