# Supplementary figures and images for: Identifying potential biomarkers for early evaluating mechanical compression injuries to skeletal muscle through proteomic analysis: A rat model
Source: PLoS One. 2025 May 27;20(5):e0324706. doi: 10.1371/journal.pone.0324706 (PMC12111613; doi:10.1371/journal.pone.0324706)

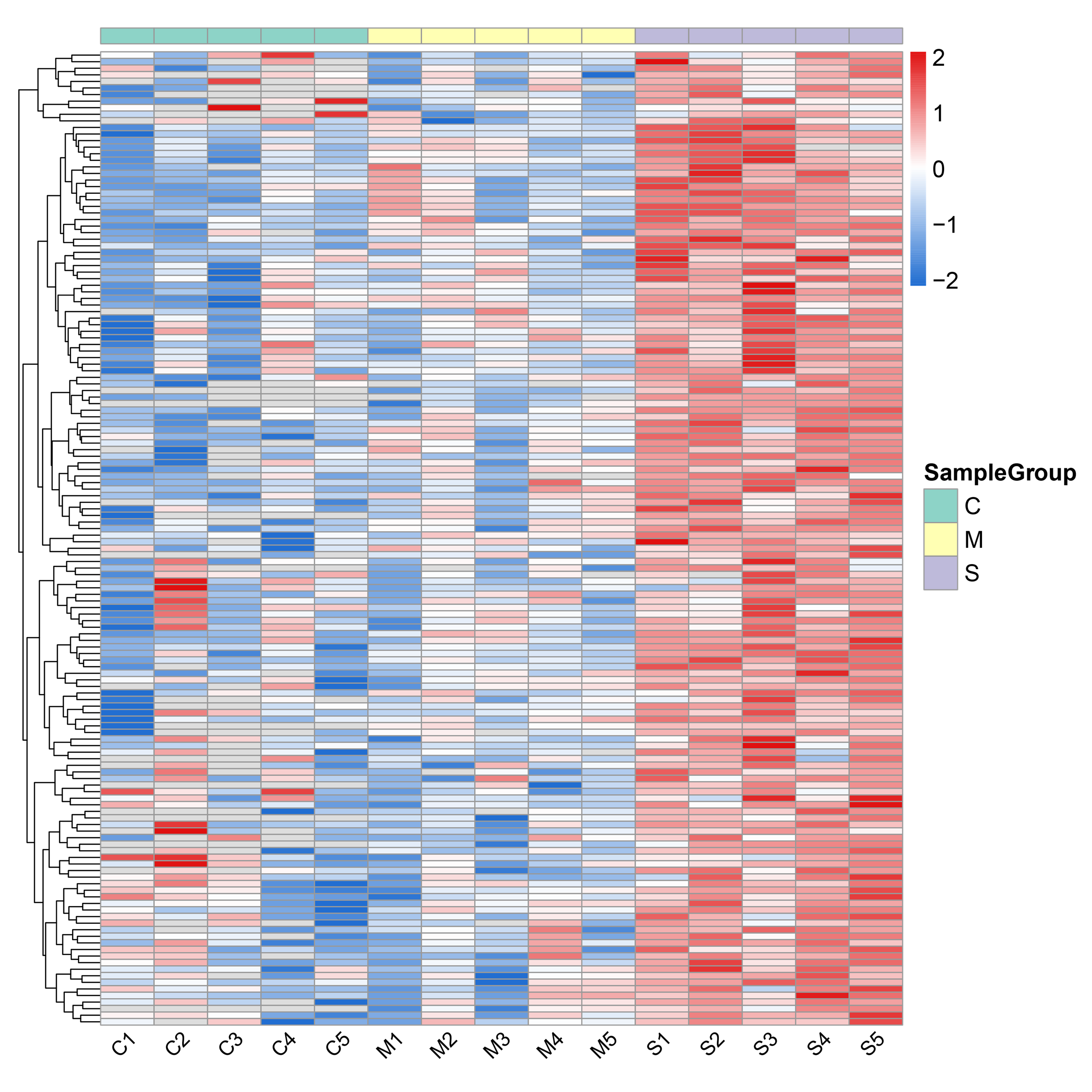

Supplement: S2 Fig — (TIF) [file pone.0324706.s002.tif]

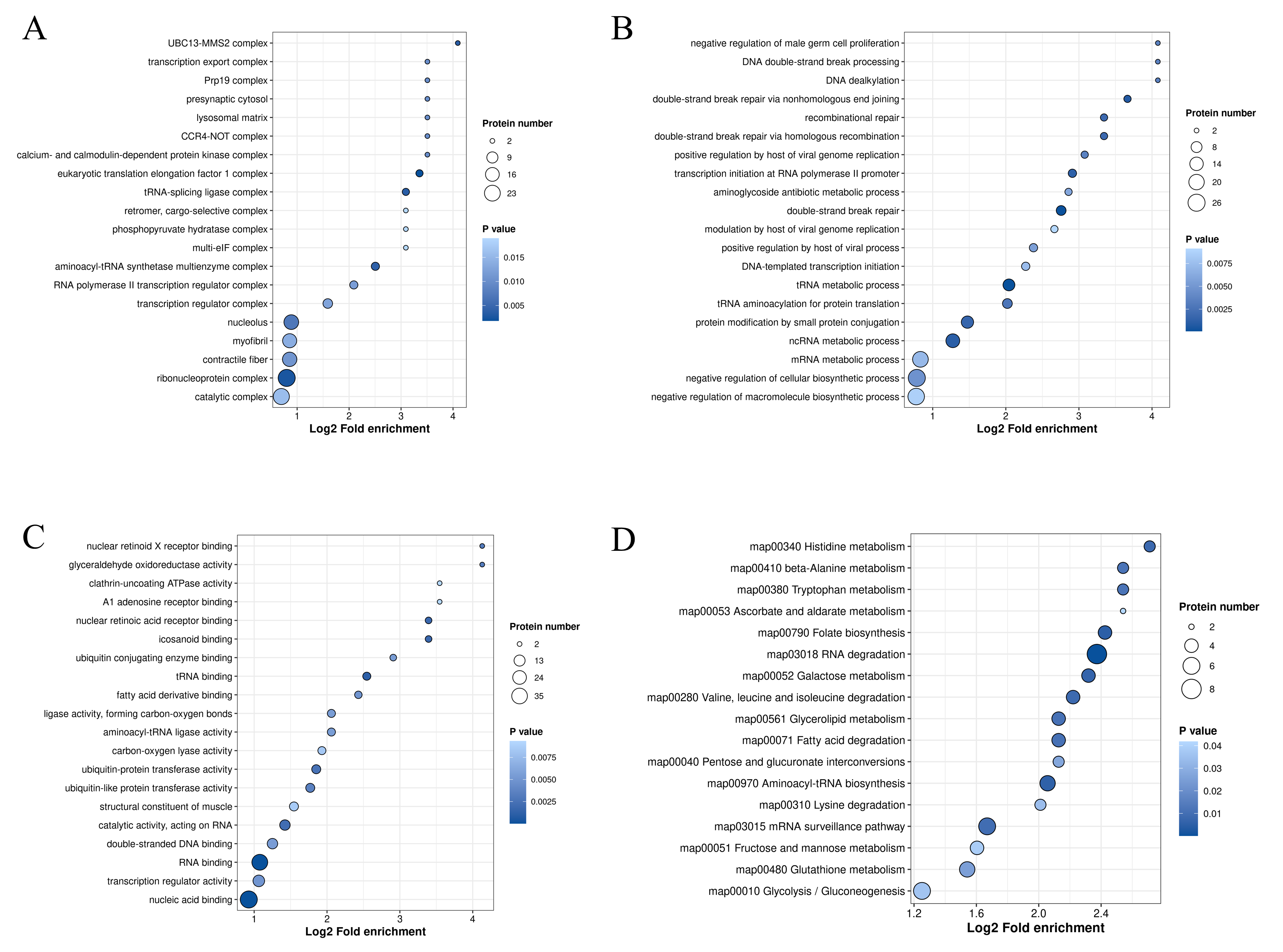

Supplement: S3 Fig — (A) Cellular component of DEPs. (B) Biological processes of DEPs. (C) Molecular function of DEPs. (D) KEGG pathways. (TIF) [file pone.0324706.s003.tif]

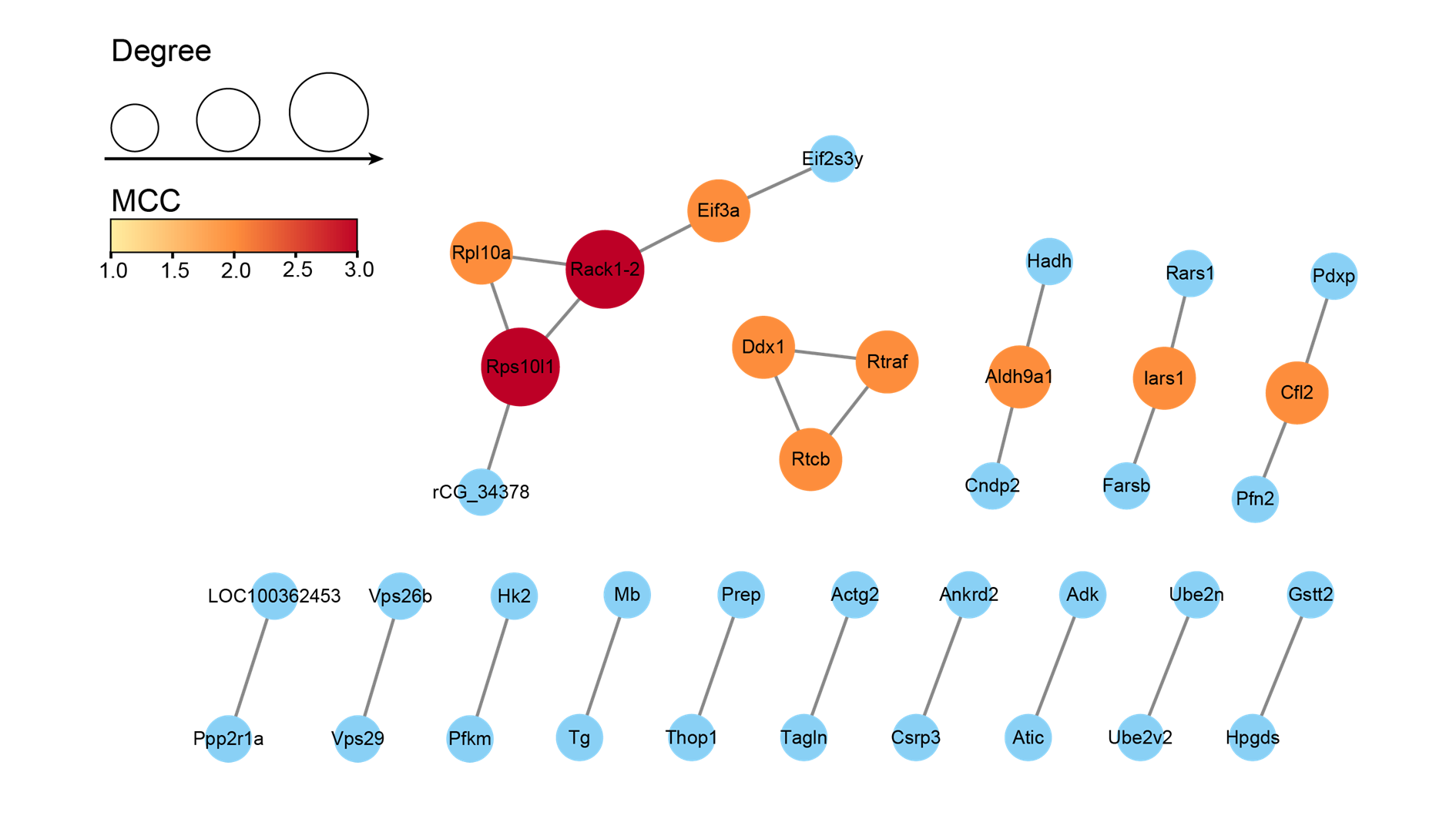

Supplement: S4 Fig — (TIF) [file pone.0324706.s004.tif]
